# Bovine and porcine heparins: different drugs with similar effects on human haemodialysis

**DOI:** 10.1186/1756-0500-6-230

**Published:** 2013-06-13

**Authors:** Ana MF Tovar, Lisandra AC Teixeira, Simone M Rembold, Maurilo Leite, Jocemir R Lugon, Paulo AS Mourão

**Affiliations:** 1 Laboratório de Tecido Conjuntivo, Hospital Universitário Clementino Fraga Filho, Universidade Federal do Rio de Janeiro, Rio de Janeiro, Brazil; 2 Instituto de Bioquímica Médica, Hospital Universitário Clementino Fraga Filho, Universidade Federal do Rio de Janeiro, Rio de Janeiro, Brazil; 3 Divisão de Nefrologia, Departamento de Medicina Clínica, Universidade Federal Fluminense, Niterói, Brazil; 4 Serviço de Nefrologia, Departamento de Clínica Médica, Hospital Universitário Clementino Fraga Filho, Universidade Federal do Rio de Janeiro, Rio de Janeiro, Brazil

**Keywords:** Heparin, Antithrombotic effects, Haemodialysis

## Abstract

**Background:**

Heparins from porcine and bovine intestinal mucosa differ in their structure and also in their effects on coagulation, thrombosis and bleeding. However, they are used as undistinguishable drugs.

**Methods:**

We compared bovine and porcine intestinal heparin administered to patients undergoing a particular protocol of haemodialysis. We compared plasma concentrations of these two drugs and also evaluated how they affect patients and the dialyzer used.

**Results:**

Compared with porcine heparin, bovine heparin achieved only 76% of the maximum plasma concentration as IU mL^-1^. This observation is consistent with the activities observed in the respective pharmaceutical preparations. When the plasma concentrations were expressed on weight basis, bovine heparin achieved a maximum concentration 1.5 fold higher than porcine heparin. The reduced anticoagulant activity and higher concentration, on weight basis, achieved in the plasma of patients under dialysis using bovine instead of porcine heparin did not affect significantly the patients or the dialyzer used. The heparin dose is still in a range, which confers security and safety to the patients.

**Discussion:**

Despite no apparent difference between bovine and porcine intestinal heparins in the haemodialysis practice, these two types of heparins should be used as distinct drugs due to their differences in structure and biological effects.

**Conclusions:**

The reduced anticoagulant activity achieved in the plasma of patients under dialysis using bovine instead of porcine heparin did not affect significantly the patients or the dialyzer.

## Background

Heparin has been used for more than 50 years to treat and prevent thrombosis. It is also required for extracorporeal circulation during haemodialysis or cardiovascular surgery [1-3]. Heparin is still obtained exclusively from animal tissues. Due to the higher anticoagulant activity of the porcine than bovine heparin [4], and the advent of the bovine spongiform encephalopathy, the use of heparins from bovine tissues has been nearly abolished in European countries and in the United States. However, there is an increasing debate about a more extensive use of heparin from bovine intestine.

In the past heparin from bovine lung was extensively used until its replacement by porcine heparin. More recently this drug has been also extracted from bovine intestine and sometime the debate is confusing referring to “bovine” heparin as the same drug, irrespectively of the tissue of origin (lung or intestine).

Porcine intestinal heparin consists mainly of the repeating trisulfated disaccharide →4-α- Ido2S-1→4-α-GlcNS6S-1→. Heparin from bovine lung has almost a similar disaccharide composition. In contrast, heparin preparations from bovine intestine are more heterogeneous and contain α-glucosamine with significant substitution variations: ~60% are *N*,6-disulfated, as in porcine heparin, while ~40% are 6-desulfated [5]. Other minor but biologically relevant differences in structure are also observed between the two intestinal heparins. In particular, *N*,3 and 6-trisulfated α-glucosamine (lower proportions) and α-Glc*N*S-1→4-β-GlcA and α-IdoA2S-1→4-α-Glc*N*Ac (higher amounts) prevail in bovine heparin [6]. Other studies also reported that bovine and porcine heparins differ in their sulfation patterns [7,8] but there are quantitative differences between our and these previous reports possibly due to the use of NMR spectrometers with different resolutions (800 *vs.* 500 MHz, respectively).

Bovine and porcine intestinal heparins differ significantly in their effects on coagulation, thrombosis and bleeding [5]. On a weight basis, bovine intestinal heparin exhibited approximately half of the anticoagulant and antithrombotic effects, but similar bleeding tendency. The doses of bovine heparin required for an effective antithrombotic protection and adverse bleeding effect are closer than those observed for porcine heparin. More recently, we demonstrated that pharmaceutical grade heparins from bovine intestine contain a mixture of glycans with multiple sulfation patterns and anticoagulant effects [6].

Differences in brand of porcine heparin used during cardiovascular bypass were associated with bleeding complications and clinical outcomes [9,10]. Even more significant the use of porcine and bovine heparins as undistinguishable drugs, besides their differences in structure and anticoagulant effects, raises important questions concerning their clinical effects on patients. Are they similar in efficacy and safety? Of course the clarification of this issue has considerable practical and conceptual implications, since bovine heparin used in some countries and banished in others. We now compare the effects of bovine and porcine heparins in patients under hemodialysis.

Currently, the population under dialysis in the world is estimated in about 1.5 million, with ~90% of them on haemodialysis [11]. In Brazil, the estimated number of patients on dialysis was around 92,000 on 2010 [12], and the proportion of patients on haemodialysis is similar to other countries. In most dialysis centers in Brazil, heparin is administered at the beginning of the dialysis session as a bolus injection, and the dose in anticoagulant units is adjusted for each patient at the beginning of the therapy. Porcine and bovine intestinal heparins are used as undistinguishable drug.

The major aim of our study is to compare bovine and porcine intestinal heparins in the course of dialysis session. Our results indicate that reduced anticoagulant activities and higher concentrations, on weight basis, were achieved in the plasma of patients under dialysis using bovine instead of porcine heparin. However, the difference between two types of heparin did not affect significantly the patients and the dialyzer used. The heparin dose was still in the range which confers security and safety to the patients.

## Methods

### Patients

Our study included 17 patients (Table 1) undergoing regular haemodialysis and using unfractionated heparin as the anticoagulant. Only patients with native fistulae were included. Patients were kept at their usual heparin dose established by the assistant nephrologist, since any change in the dose to attend the study purposes would be ethically unacceptable. As a result, the doses among patients varied ~28% (Table 1). However, when switching from one type of heparin to another, the dose was maintained at the same anticoagulant units used for each patient. One single batch of pharmaceutical preparation of bovine and porcine heparin was used. Blood lines and dialyzers were filled with saline without heparin. Heparin was administered *in bolus* through the venous needle 5 min before the start of the dialysis session. All patients were treated with HF-80® dialyzers (high flux polyssulfone, 2.1 m^2^ surface area, Fresenius AG, Bad Homburg, Germany). Dialyzers were reprocessed automatically using hydroxide peroxide/per acetic acid mixture and discharged in case of internal cell volume lower than 80% of the initial volume.

**Table 1:** General characteristics of the study population

N	17
Gender (M/F)	10/7
Age, years	45 ± 12^a^
Skin color (Black/Mulatto/White)	5/7/5
Primary renal disease	
Hypertensive nephropathy	9
Diabetic nephropathy	3
Other	4
Unknown	1
Dialysis vintage (months)	53 ± 39
Heparin dosage (reported IU.kg^-1^ body weight)	141 ± 41

^a^mean ± SD.

### 1D ^1^H NMR analysis of the heparin preparations

The pharmaceutical preparations of bovine and porcine heparin used in this study were analyzed by one-dimensional (1D) ^1^H NMR spectra, as described [5,13]. Approximately 0.4 mL of the pharmaceutical preparations, containing ~10 mg of heparin, were lyophilized and dissolved in 0.5 mL of 99.9% deuterium oxide (Cambridge Isotope Laboratory, Cambridge, MA, USA). The spectra were recorded using a Bruker DRX 800 MHz apparatus with a triple resonance probe, as described previously. All spectra were recorded at 35°C with HOD (deuterated water exhibiting a peak due to exchange with residual H_2_O) suppression by presaturation. Chemical shifts are displayed relative to external trimethyl-silylpropionic acid at 0 ppm for ^1^H and relative to methanol for ^13^C.

### Determination of the plasma concentration of heparin as IU mL^-1^

In order to determine the plasma concentration of heparin, as IU mL^-1^, 30 μL of plasma collected from the patients, mixed with 70 μL of normal human plasma was incubated with 100 μL of aPTT reagent (kaolin bovine phospholipid reagent from Biolab-Merieux AS, Rio de Janeiro, Brazil). The dilution of the patient plasma with normal human plasma was necessary in order to reduce the clotting time in the presence of the high concentration of heparin, administered during the dialysis session. Similar limitation for the use of undiluted plasma to monitor plasma concentration of heparin was previously reported [14]. After 2 min of incubation at 37°C, 100 μL of 25 mM CaCl2 was added to the mixtures, and the clotting time was recorded in a coagulometer (Amelung KC4A; Heinrich Amelung GmbH, Lemgo, Germany). In parallel, various concentrations of the 5th International Standard for Unfractionated Heparin (97/578, 229 IU mg^-1^) obtained from the National Institute for Biological Standard and Control (UK) were mixed with 100 μL of human plasma and the clotting time recorded as described above. The data obtained fit a polynomial function, according to the equation:

$$\left[T/T_{o}\right]=a+b_{1}.\left[\mathrm{IU}\right]+b2.{\left[\mathrm{IU}\right]}^{2},$$

where [T/T_o_] represents the ratio of clotting time in the presence (T) or absence (T_o_) of different heparin concentrations, [IU] the amounts of anticoagulant activity, a is the intersection of the curve in the y axis, which is equal 1, b_1_ and b_2_ are constant values derived from the polynomial fit. Based on this equation we can derive the amounts of anticoagulant activity in 30 μL of patient plasma and obtain the value as IU mL^-1^. The plasma concentration of heparin assessed by aPTT assay strongly correlates with anti-Xa activity and other coagulation assays [14].

### Determination of the plasma concentration of heparin as μg mL^-1^

The concentrations of heparin on the pharmaceutical preparations of bovine and porcine heparin were estimated based on their hexuronic acid content [15]. In order to determine the plasma concentration of heparin as μg mL^-1^, we perform calibration curves of T/T_o_*versus* concentration of heparin as μg of hexuronic acid. Again the data fit a polynomial function, according:

$$\frac{T}{T_{o}}=a+b_{1}\left[\mu g\right]+b_{2}{\left[\mu g\right]}^{2},$$

as described above for determination of heparin concentration based on the anticoagulant activity but now expressing the results as μg hexuronic acid mL^-1^, that is the amount of heparin on weight basis.

### Anticoagulant activities of the pharmaceutical preparations of bovine and porcine heparins

The anticoagulant activities of the pharmaceutical preparations were checked based on the aPTT assays using as standard the 5^th^ International Standard for Unfractionated Heparin, as described [5].

### Statistical analysis

Statistical analysis was undertaken by Sigma Stat software (Systat, San Diego, CA, USA) employing the Mann–Whitney Rank Sum Test. P-value of <0.05 was considered as statistically significant.

## Results and discussion

**Figure 1:**
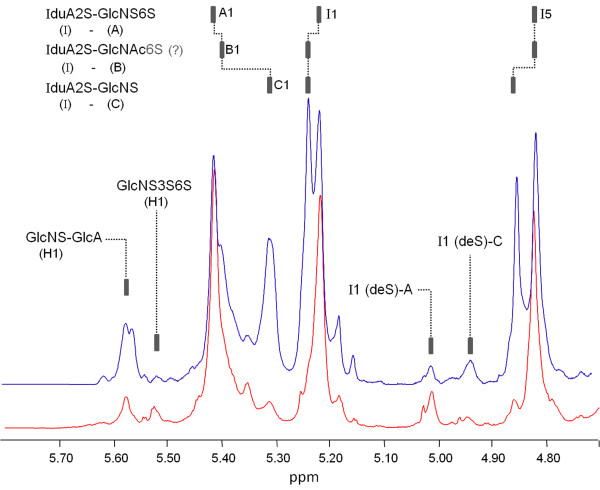
**Analysis of the pharmaceutical preparations of bovine (in blue) and porcine (in red) heparin by 1D **^**1**^**H NMR spectroscopy at 800 MHz.** The signals designated as A1 correspond to H1 of *N*,6-disulfated α-glucosamine units; B1 and C1 to H1 of *N*-acetylated and 6-desulfated α-glucosamine units, respectively. These chemical modifications of the α-glucosamine units shift ~0.2 ppm downfield H1 of the neighbor 2-sulfated α-iduronic acid (I1 in the panel). Furthermore, 6-desulfation but not *N*-acetylation of the α-glucosamine units shift ~0.2 ppm downfield H5 of the same α-iduronic acid units (I5 in the panel). Glc*N*S-GlcA corresponds to H1 of *N*-sulfated α-glucosamine linked to β-glucuronic acid and GlcNS3S6S to H1 of *N*,3,6-trisulfated α-glucosamine units. I1 (deS)-A and I1(deS)-C are H1 of desulfated α-iduronic acid residues linked to *N*,6-disulfated and *N*-sulfated α-glucosamine units, respectively.

The pharmaceutical preparations of bovine and porcine heparin (one single batch of each one) employed in this study were initially analyzed by 1D ^1^H NMR spectroscopy at 800 MHz (Figure 1). Structural differences can be clearly observed between them. Several additional ^1^H NMR signals are observed in bovine heparin due mostly to 6-desulfation and *N*-acetylation of the α-glucosamine units (compare signals in blue and red in Figure 1. Approximately, 400 batches of bovine and porcine heparins available for clinical use in Brazil were previously analyzed in our laboratory and showed consistently the same characteristic differences in structure.

Display of the ^1^H NMR spectra of the two pharmaceutical preparations employed in this study (Figure 1) assures a clear correlation between heparin structures and their effects on dialysis sessions. Bovine heparins obtained from other producers may differ in their sulfation pattern. This aspect is especially relevant since pharmaceutical preparations of bovine heparins contain mixtures of low and high sulfated fractions [6].

**Figure 2:**
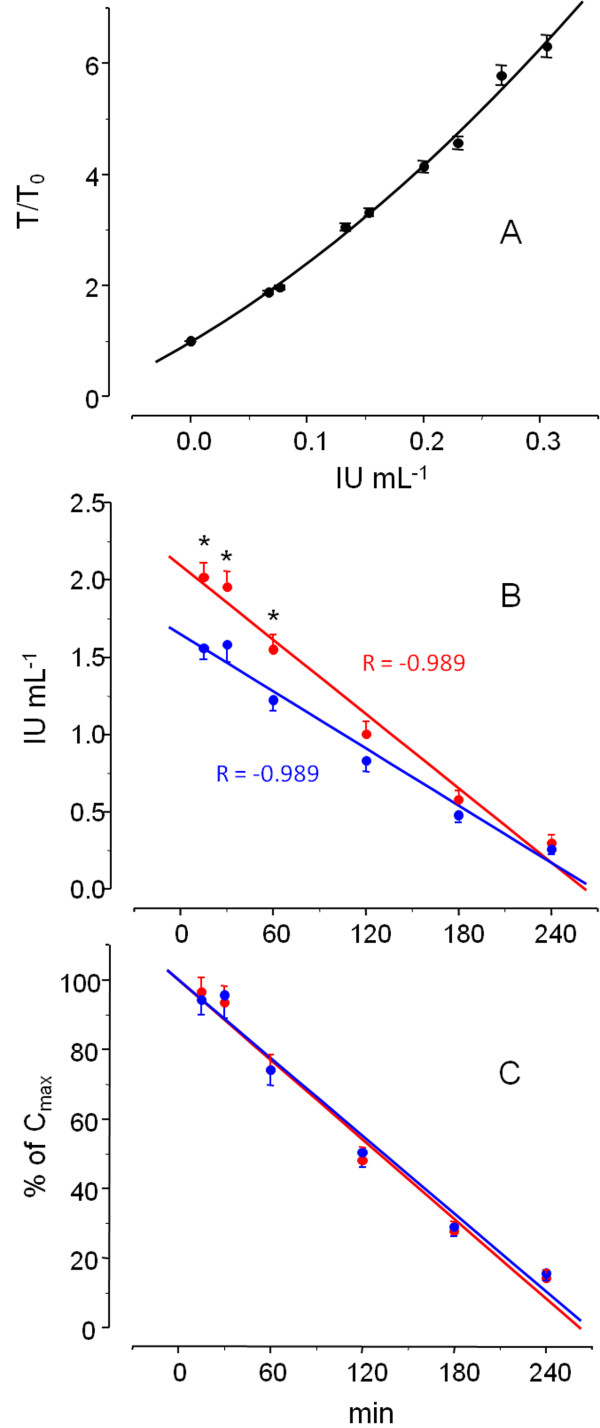
**Plasma concentrations of bovine and porcine heparins. A:** Standard curves obtained with the 5^th^ International standard of unfractionated heparin used to determine the plasma concentrations of heparin as IU mL^-1^. The results are expressed as mean±SE from 60 different standard curves used during the study. **B** and **C:** plasma concentration of bovine (in blue) and porcine (in red) heparins as IU mL^-1^ (**B**) and as % of C_max_ (**C**). See description of the assay in the Method section. The results in B and C are expressed as mean±SE from 49 and 41 dialysis sessions with bovine and porcine heparin, respectively. The data fitted a linear correlation using a Microcal Origin PC program and the values of linear correlation coefficients (R) are indicated in the panel. In Panel **B**, * indicates *p*<0.02 for bovine *vs.* porcine heparin.

These two heparin preparations were administered to the patients as a bolus injection at a dose 141 ± 41 IU (as reported anticoagulant activity) kg^-1^ body weight. Plasma samples were collected at different time points and heparin concentrations determined as IU mL^-1^ of plasma using the assay shown in Figure 2, Panel A (see also Methods).

Compared with porcine heparin, bovine heparin achieved only 78% of the maximum plasma concentration (C_max_), as IU mL^-1^ (1.65 *vs.* 2.10 IU mL^-1^, blue *vs.* red signals in Figure 2B, see data for the individual patients in Table 2, 1.63 ± 0.44 *vs.* 2.15 ± 0.56 IU mL^-1^, P < 0.01).

**Table 2:** **Number of dialysis sessions, average of the maximum plasma concentration (C**_**max**_**) as IU mL**^**-1 **^**or μg mL**^**-1 **^**(as hexuronic acid content) and t**_**½ **_**(as min) for each individual patient**

		**Porcine**				**Bovine**			
**Patient**	**Dose of heparin**	**Number of sessions**	**C**_**max **_**in plasma**		**t**_**½**_	**Number of sessions**	**C**_**max **_**in plasma**		**t**_**½**_
	**IU x 10**^**3**^		**IU mL**^**-1**^	**μg mL**^**-1**^	**Min**		**IU mL**^**-1**^	**μg mL**^**-1**^	**min**
1	12	3	2.53	3.47	141.0	3	2.57	7.08	160.9
2	9	3	2.62	3.59	144.8	3	2.01	5.54	151.7
3	12	2	2.66	3.65	126.7	3	1.77	4.88	142.3
4	10	2	2.59	3.55	168.5	2	1.69	4.66	136.1
5	9	1	2.14	2.94	159.3	2	1.54	4.24	152.8
6	11	2	1.73	2.37	105.2	3	1.06	2.92	129.1
7	6	3	1.95	2.67	127.0	3	1.35	3.72	116.2
8	7	3	1.91	2.62	138.9	2	1.24	3.42	145.8
9	10	3	1.52	2.08	111.6	3	1.01	2.78	117.5
10	9	3	1.31	1.80	112.3	4	1.26	3.47	118.7
11	8	3	1.52	2.08	116.1	3	1.69	4.66	124.0
12	9	3	2.32	3.18	148.0	3	2.10	5.79	146.1
13	8	2	3.18	4.36	142.6	3	2.26	6.23	151.9
14	5	3	1.36	1.87	110.8	3	1.23	3.39	133.5
15	12	2	2.23	3.06	128.1	3	1.91	5.26	123.7
16	12	3	2.82	3.87	129.5	3	1.69	4.66	134.4
17	10	-	-	-	-	3	1.27	3.50	133.6
Total		41	2.15±0.56	2.95±0.77	131.3±18.3	49	1.63±0.44	4.48±1.22	136.4±13.8

The time required to reduce C_max_ of heparin by 50% (t_½_) was similar for bovine and porcine preparations (133.9 and 131.0 min, respectively). This similarity is clearer when the results are expressed as percentage of C_max_*vs.* period of time after drug administration (Figure 2C, see also Table 2 for individual data).

**Figure 3:**
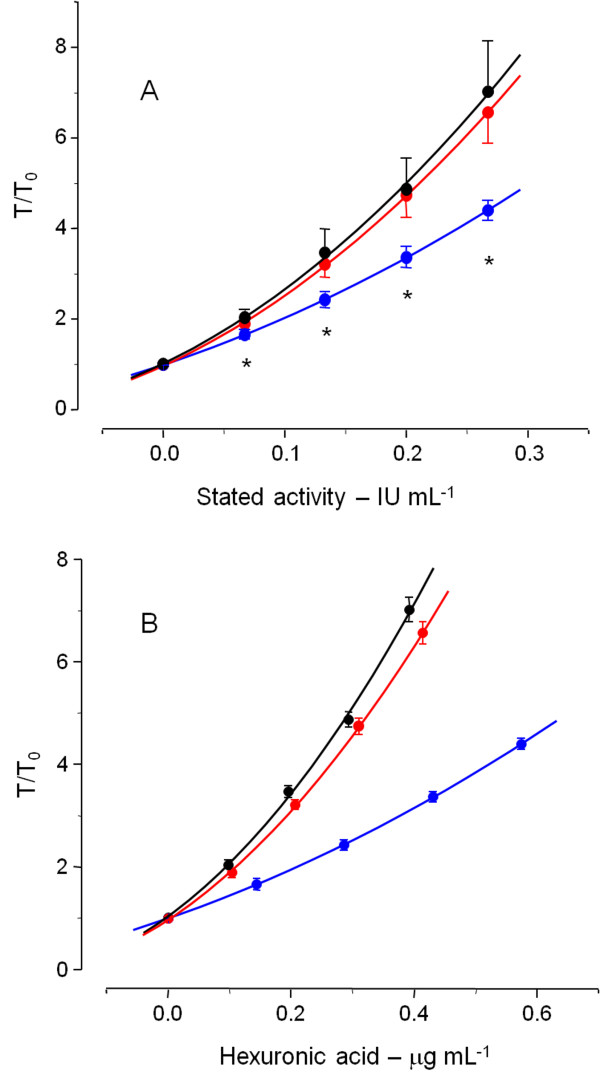
**The anticoagulant activities of the pharmaceutical preparations of bovine (in blue) and porcine (in red) heparins and of the 5**^**th **^**International Standard for Unfractionated Heparin (in black) were checked based on the aPTT assays.** (**A**) The results are expressed as IU of the anticoagulant activity reported by the manufacturer *vs.* ratio of the coagulation time in the presence (T) and in the absence of heparin (T_0_) as mean ± SD. * indicates *p* < 0.001 for bovine *vs.* porcine heparin. (**B**) The results are expressed as hexuronic acid content of the pharmaceutical preparations *vs.* ratio of the coagulation time in the presence (T) and in the absence of heparin (T_0_) as mean ± SD.

We would expect that patients shifting from one to another type of heparin should achieve similar levels of plasma anticoagulation (as IU mL^-1^). Results reported in Figure 2 and Table 2 may be interpreted as lower patient responses to bovine compared to porcine heparin, perhaps as a consequence of different bioavailability, or as differences in the activities of the respective pharmaceutical preparations. This aspect was investigated in the experiment of Figure 3. The anticoagulant activities of the pharmaceutical preparations of bovine and porcine heparins were checked by the aPTT assay (blue *vs.* red signals, respectively, in Figure 3A). Surprisingly, bovine heparin was proven to possess only ~70% of the activity reported by the manufacturer. Similar result was observed for the anti-IIa and anti-Xa activities (not shown). Indeed the final product of bovine heparin contains higher amounts on weight basis than porcine heparin (10.75 *vs.* 7.75 as mg of hexuronic acid mL^-1^, respectively), but not sufficient to compensate the stated activity.

The current approach differs substantially from that used in our previous publications [5,6]. We now use the final pharmaceutical products of bovine and porcine heparins instead of batches of their active ingredients. Reduced anticoagulant property of bovine heparin requires addition of high amounts to their final pharmaceutical products in order to achieve the same stated activity as porcine heparin (5,000 IU mL^-1^).

When the anticoagulant activities of the pharmaceutical preparations were express *versus* amounts of heparin as hexuronic acid content (μg mL^-1^), we noted that, bovine has approximately half activity of porcine heparin (Figure 3B), as we observed previously [5,6]. These standard curves were used to determine the plasma concentrations on weight basis. Clearly, bovine heparin achieved a C_max_ 1.5 fold higher than porcine heparin (4.48 ± 1.22 *vs.* 2.95 ± 0.77 μg mL^-1^, Table 2).

This data suggests that higher amounts of bovine heparin (on weight basis) are cleared from plasma at a period of time similar to that required for porcine heparin. The clearance seems to be due to a non-saturated mechanism of disappearance under the concentration range used. Of course, it is still necessary to compare the pharmacodynamic of bovine and porcine heparins in patients with normal renal function in order to evaluate the renal clearance of these two heparins.

**Figure 4:**
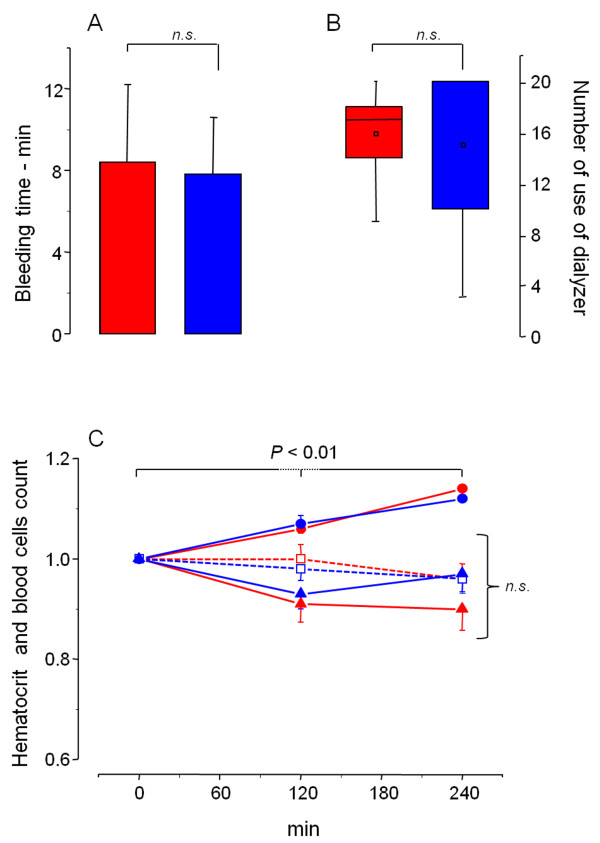
**Comparison between the effects of bovine (in blue) and porcine heparin (in red) in the bleeding time (A), number of use of the dialyzer (B) and hematocrit, leukocytes and platelet counts (C).** (**A**) Bleeding time after dialysis session, as mean ± SD. (**B**) The number of uses of the dialyzers per patient, as median (CI 95%) and mean (□). Each patient is allowed to use the dialyzer for a maximum of 20 sessions. However, at the end of each session the internal diameter of the dialyzer fibers is check. If it dropped to below 80%, the dialyzer is replaced. (**C**) Hematocrit (●), leukocytes (□) and platelet (▲) counts along dialysis sessions. The results are expressed as ratios (mean ± SE) between values observed at 120 or 240 min *vs.* values before the sessions. Leukocytes and platelet ratios were also corrected based on the hematocrit ratios to avoid the effect of hemoconcentrations (P<0.01) observed during dialysis session.

After demonstrating that higher plasma concentration (on anticoagulant basis) was achieved when porcine instead of bovine heparin was administered to the patients, the next question to address was whether this difference affects either the patients or the dialyzer. This aspect was investigated by three parameters: the bleeding time at the end of the dialysis session (Figure 4, Panel A), the number of uses of the dialyzer per patient (Panel B) and the blood cells count along the dialysis session (Panel C). Based on these parameters, no significant differences were observed between bovine and porcine heparins (Table 3). Furthermore, no other clinical feature was observed during the 680 dialysis sessions included in the study, using either porcine (340) or bovine (340) heparins.

**Table 3:** **Hematocrit, leukocytes and platelet counts along the dialysis sessions with blood cells count along dialysis session with porcine *vs. *bovine heparin**

		**Heparin**	
	**Time**	**Porcine**	**Bovine**
	**min**	**mean ± SD**	
Hematocrit (%)	0	35.7 ± 3.1 (1.00) *	36.7 ± 4.5 (1.00) *
	120	37.9 ± 3.4 (1.06 ± 0.05) *	39.2 ± 4.0 (1.07 ± 0.11) *
	240	41.0 ± 4.0 (1.15 ± 0.08) *	40.9 ± 4.7 (1.12 ± 0.10) *
Leukocytes (cells mm^-3^ 10^-3^)	0	7.6 ± 2.2 (1.00) *	7.4 ± 2.2 (1.00) *
	120	7.9 ± 2.0 (1.00 ± 0.17)**	7.6 ± 2.0 (0.98 ± 0.15)**
	240	8.1 ± 2.1 (0.96 ± 0.18)**	7.7 ± 2.0 (0.96 ± 0.18)**
Platelet (count mm^-3^ 10^-3^)	0	217.3 ± 70.5 (1.00) *	220.6 ± 73.1 (1.00) *
	120	209.5 ± 64.8 (0.91 ± 0.18)**	216.6 ± 62.4 (0.93 ± 0.18)**
	240	222.1 ± 68.9 (0.96 ± 0.18)**	232.3 ± 60.6 (0.98 ± 0.22)**

*Ratios of values *vs*. values before heparin administration.

**Ratios of leukocytes or platelet at 120 or 240 min *vs*. values before heparin administration corrected to the hematocrit variation.

The reduced anticoagulant activity and higher concentration, on weight basis, achieved in the plasma of patients under dialysis using bovine instead of porcine heparin did not affect significantly the patients or the dialyzer used. The heparin dose is still in a range, which confers security and safety to the patients.

Despite this observation, our results must be interpreted carefully. Heparin is mostly used to treat and prevent thromboembolic diseases. Can we assure that a ~30% reduced anticoagulant activity achieved with pharmaceutical preparations of bovine heparin will guarantee similar protection or efficiency as porcine heparin? In the case of cardiovascular surgeries, higher doses of heparin are used as compared to dialysis sessions. Hence, studies designed for these procedures in which higher doses of heparin are employed are important to be performed. In this case, can we assure that removal of heparin from plasma is non-saturated with bovine heparin, where significantly higher doses of this glycosaminoglycan are required for anticoagulation? Residual heparin may have severe consequences in these patients, such as bleeding and other side effects. Certainly, these aspects require careful investigation. Remarkable, high rates of bleeding episodes were observed among Brazilian patients during cardiovascular surgeries, when bovine heparin replaced porcine heparin [16].

Two brands of porcine heparin were compared in patients submitted to bypass surgery. Differences in the postsurgical outcomes were not associated with variation in the anticoagulant activity of the two pharmacological preparations but with a particular brand [9,10]. It is still unclear the particular aspect of the heparin preparation which confer the less favorable outcome after bypass surgery.

Up to the 80s’, heparins obtained from bovine lung (instead of bovine intestine) were largely employed [4,17]. The structures of heparins from bovine lung and porcine intestine are closely related and both differ significantly from bovine intestinal heparin. This type of heparin was just recently available for clinical use and restricted to a few countries. We believe its use requires a detailed analysis to assure its safety and efficiency under the variety of clinical events where the drug could be used. In conclusion, despite no apparent difference between bovine and porcine intestinal heparins in the haemodialysis practice, these two types of heparins should be used as distinct drugs due to their differences in structure and biological effects.

## Competing interests

The authors declare that they have no competing interests.

## Authors’ contributions

AMFT planned and interpreted the anticoagulant assays and drafted the manuscript. LACT carried out the anticoagulant assays. SMR collected samples and performed assays. MLJr helped to plan the study. JRL designed the study and drafted the manuscript. PASM collected the NMR data and wrote the manuscript. All authors read and approved the final manuscript.
